# Rupture of Abdominal Aortic Aneurysm Caused by Combined Type IIIb and Type Ia Endoleak with the Endurant II Endograft: A Case Report

**DOI:** 10.3400/avd.cr.20-00175

**Published:** 2021-06-25

**Authors:** Takanobu Okazaki, Masaki Hamamoto, Taiichi Takasaki, Keijiro Katayama, Taira Kobayashi, Shinya Takahashi

**Affiliations:** 1 Department of Cardiovascular Surgery, JA Hiroshima General Hospital, Hatsukaichi, Hiroshima, Japan; 2 Department of Cardiovascular Surgery, Hiroshima University Hospital, Hiroshima, Hiroshima, Japan

**Keywords:** abdominal aortic aneurysm, endovascular aneurysm repair, type IIIb endoleak

## Abstract

We report a case of combined types IIIb and Ia endoleak that developed 6 years after endovascular aneurysm repair (EVAR) with the Endurant II® endograft for abdominal aortic aneurysm (AAA). The patient presented with post-EVAR AAA rupture and underwent emergency open repair. We observed types IIIb and Ia endoleak and successfully performed felt banding to preserve the stent graft. Type IIIb endoleak with the Endurant® endograft is rare, and treatments have not been fully established. We summarized the case reports regarding type IIIb endoleak with the Endurant® endograft and mainly discussed the treatments.

## Introduction

Endovascular aneurysm repair (EVAR) has been widely performed to treat abdominal aortic aneurysm (AAA). EVAR has the advantages of faster postoperative recovery and lower hospital mortality compared with open repair. On the other hand, various complications, including endoleak, migration, and infection, have been reported with the prolonged observation period after EVAR. The majority of additional treatments, endovascular or open repair, have been performed to treat endoleak. In this study, we report a rare case of combined types IIIb and Ia endoleak with the Endurant II® stent graft. The endoleaks developed AAA rupture and were successfully treated using felt banding. We discussed the possible mechanism of combined endoleaks and the surgical procedures for type IIIb endoleak.

## Case Report

**Figure 1:**
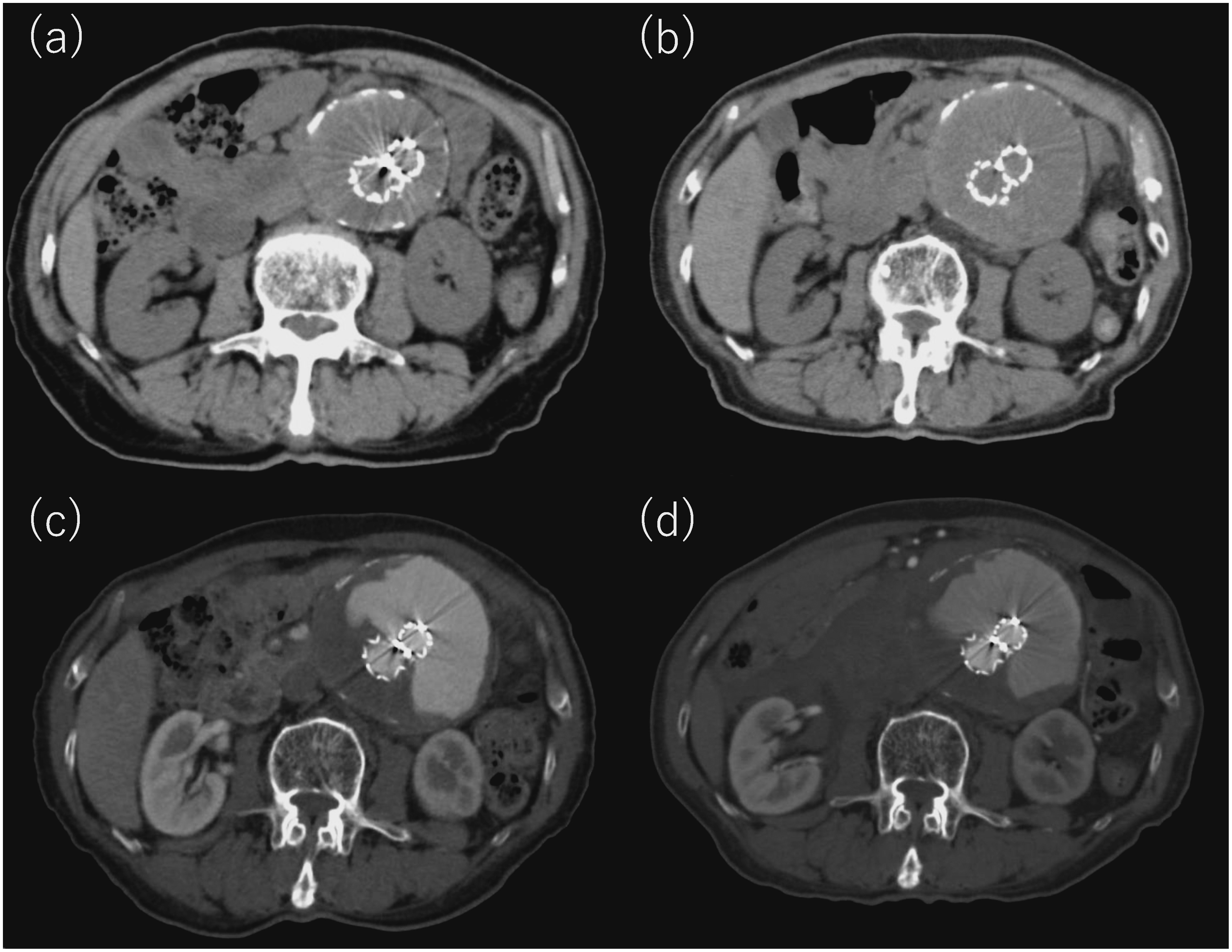
Fig. 1 Abdominal computed tomographic scan demonstrated. (**a**) No progressive dilatation of the aneurysm with its diameter of 60 mm, (**b** and **c**) aneurysmal dilatation of up to 75 mm in diameter and endoleak around the left leg, (**d**) aneurysm rupture.

An 85-year-old man with hypertension, stroke, and severe asthma underwent EVAR with the Endurant II® endograft (Medtronic, Santa Rosa, CA, USA) and right internal iliac coiling for the treatment of a 67-mm infrarenal AAA and a 23-mm internal iliac aneurysm at the other hospital. EVAR had terminated with no sign of endoleak by completion angiography. Non-enhanced computed tomographic (CT) scan during yearly outpatient follow-up also showed no progressive dilatation of the aneurysm (**Fig. 1a**).

About 6 years later, however, CT scan showed rapid dilatation of the aneurysm with the diameter from 60 to 75 mm in a year. The contrast medium was filled inside the aneurysmal sac, suggesting the presence of type IIIa endoleak (**Figs. 1b** and **1c**). We planned an elective open surgery to treat the endoleak. Unfortunately, sudden abdominal pain and loss of consciousness occurred, and urgent CT showed ruptured AAA (**Fig. 1d**). He underwent emergency open surgery.

**Figure 2:**
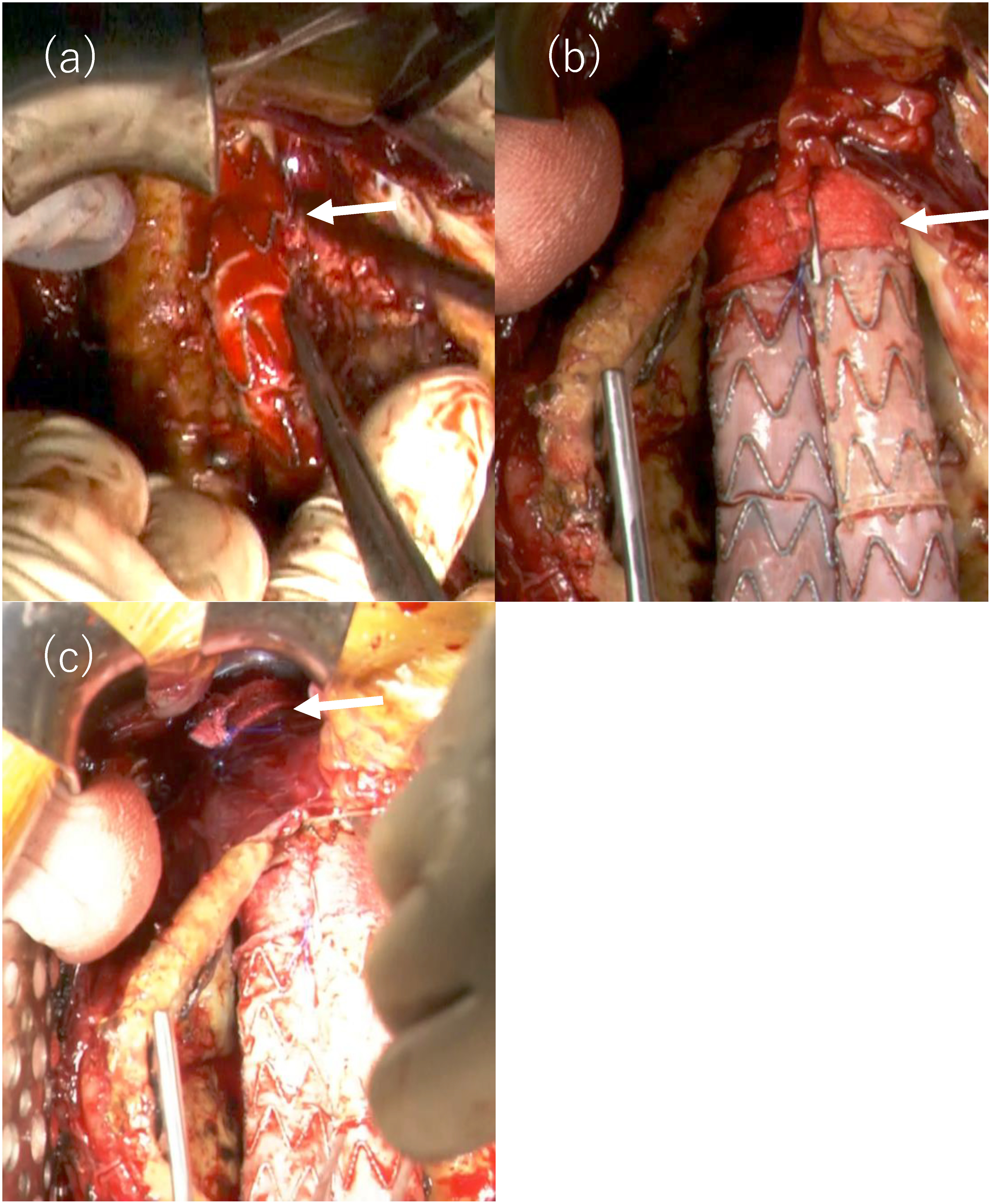
Fig. 2 Intraoperative photo showed. (**a**) Type IIIb endoleak (white arrow) at the main body of the Endurant II® endograft, (**b**) direct banding using a Teflon felt strip (white arrow) around the main body covering a type IIIb endoleak, (**c**) aortic banding (white arrow) for the treatment of type Ia endoleak.

We made a median laparotomy and found retroperitoneal hematoma with small amount of bloody ascites. Epiaortic echo showed no blood flow within the aneurysm. We punctured the aneurysm with an 18-gauge needle to measure the intra-aneurysmal pressure, which showed approximately 25 mmHg with no pulsatile flow. We assumed that there was no substantial blood flow into the aneurysm through the endoleak. The aneurysm was incised with a Teflon tape around the neck being pulled up without aortic clamp and heparin administration. When the mural thrombus was removed, active blood oozing from the fabric was observed on the left posterolateral side and just above the flow divider of the main body, which was considered to be type IIIb endoleak (**Fig. 2a**). When traction of the tape around the aortic neck was loosened, blood flow was observed from the proximal side, which was type Ia endoleak. Type IIIb endoleak from the fabric was treated using a Teflon felt strip (15 mm wide and 1.8 mm thick) being directly wrapped around the stent graft and tightly banded (**Fig. 2b**). Proximal aortic neck was also banded with the same felt strip as the treatment for type Ia endoleak (**Fig. 2c**). Aortic wall was tightly sutured to cover the stent graft. The operation time was 260 min.

Postoperative enhanced CT scan revealed no endoleak and no further dilatation of the aneurysm. He was discharged home on postoperative day 29 without any aortic complications.

## Discussion

The number of patients requiring open surgical conversion and secondary endovascular intervention after EVAR has been increasing.^1,2)^ The rate of open conversion after EVAR ranges from 2.2% to 2.5%^1,3)^ and perioperative mortality from 6.2% to 9.9%, which is significantly higher than that of the initial open surgery.^2)^ The most common cause of open conversion is endoleak, accounting for approximately 80% of all open conversion cases.^1)^ The majority of the endoleaks leading to open conversion are types I and II endoleak. In contrast, the frequency of type III endoleak is low at the rate of 2.1%.^4)^ In particular, type IIIb endoleak from a fabric tear has been reported even less frequently, with only a few case reports on the Endurant® and Endurant II® endografts (**Table 1**).^5–9)^ Type IIIb endoleaks were detected at an average of 28 months after EVAR, with three of five cases developing ruptured AAA. The common location of type IIIb endoleak was the main body at the level of the contralateral limb. The causes of type IIIb endoleak included a fabric tear resulting from repeat abrasion between the main body and the proximal contralateral limb or calcified aortic wall and excessive inflation of a touch-up balloon at the initial EVAR. As for the treatments, two of six patients underwent open repair with partial removal of the stent graft followed by graft replacement. The other patients underwent catheter-based procedures, including aorto-uni-iliac stent graft concomitant with femorofemoral crossover bypass and endovascular relining of the stent graft.

**Table 1:** Table 1 Characteristic of type IIIb endoleak with Endurant® and Endurant II® endografts

Author	Age at EVAR (year)	Brand of endoprosthesis	Event	Time interval from EVAR to diagnosis	Location of type IIIb endoleak	Treatment	Outcome
McWilliams	86	Endurant	Rupture	48 months	The main body at the level of the contralateral limb	Open conversion (graft replacement)	Alive
	76	Endurant	Expansion	48 months	The main body at the level of the contralateral limb	Relining endograft	Alive
Barburoglu	81	Endurant	Rupture	14 months	The main body endograft	Relining endograft	Dead
Abouliatim	81	Endurant	—	4 days	The main body at the level of the contralateral limb	Relining endograft	Alive
Koizumi	74	Endurant II	Rupture	6 months	The main body and the leg	Relining endograft	Alive
Kobayashi	83	Body, Endurant Leg, Excluder	Expansion	24 months	The main body	Open conversion (graft replacement)	Alive
*our case*	85	Endurant II	Rupture	79 months	The main body at the level of the contralateral limb	Open conversion (graft replacement)	Alive

EVAR: endovascular aneurysm repair

In our case, preoperative CT scan showed contrast medium filling inside the aneurysm near the legs, suggestive of type IIIa endoleak. In fact, type IIIb endoleak from the fabric tear within the main body was observed. We hypothesized that the proximal edge of the contralateral leg impinged on the main body and the fabric tear eventually developed. Furthermore, type Ia endoleak was also found during the open repair in our case. Endoleak is commonly detected as one isolated type. As the potential mechanism of combined endoleaks, type Ia endoleak may be attributable to progressive aneurysmal dilatation and proximal neck recontouring induced by the type IIIb endoleak. In addition, dissecting maneuver around the aortic neck could cause migration of the stent graft.

Open surgery after EVAR can be performed with or without partial or complete removal of a stent graft. There is no significant difference in operative mortality between the removal and preservation of a stent graft, but stent graft removal has significantly longer operative time.^2)^ Stent graft removal followed by graft replacement requires the selection of appropriate aortic clamp site to remove the graft partially or completely without avoidance of excessive stress to the aorta and stent graft. On the other hand, stent graft preservation carries some risks of graft infection and new development of type IIIb endoleak accompanied by the fabric deterioration. Based both on the evidence and deteriorated physical conditions related to rupture, we decided to select the preservation of the stent graft instead of the removal and replacement of the stent graft. The preserved graft needs to be postoperatively observed using enhanced CT scan for the detection of endoleaks. Graft replacement, therefore, seemed to be preferred over graft preservation in patients with difficulty in undertaking enhanced CT scan because of comorbidities.

An adjunctive technique of external aortic banding has been used to secure the proximal neck and reinforce the aortic wall for the treatment of type I endoleak. Krajcer et al. have reported that aortic banding was a safe, effective, and low-cost procedure with good long-term results.^10)^ In contrast, management for type IIIb endoleak has not been established. In our case, type IIIb endoleak was intraoperatively identified and treated using direct banding with a thick felt strip as is the treatment for type Ia endoleak. We were able to easily control blood oozing. Controlling type IIIb endoleak by direct banding was considered to be difficult because the nitinol exoskeletal structure of the Endurant II® endograft could produce a small gap between the felt strip and fabric. To address this concern, we used a 1.8-mm-thick felt strip and tightly wrapped it around the endoleak lesion. This sentence was edited for clarity. Please check if the revision conveys your intended meaning. The exoskeletal structure, however, could prevent the felt strip migration because of increased resistance between the felt strip and graft. Another procedure for type IIIb endoleak is a simple suture closure of the fabric tear. In our case, the fabric tear was located at the left posterolateral side of a stent graft with a nitinol exoskeletal structure. These conditions, the bleeding lesion and the exoskeletal structure, made it difficult to directly suture the fabric tear. The other risk was suture hole bleeding. The patient should be carefully observed because there have been no reports on the long-term results of felt banding for type IIIb endoleak.

## Conclusion

We experienced a case of combined type IIIb endoleak from a fabric defect and type Ia endoleak 6 years after EVAR using the Endurant II® endograft, which led to aneurysm rupture. We successfully treated type IIIb endoleak using direct banding with a thick felt and type Ia endoleak using external aortic banding.
